# Combined high dose radiation and pazopanib in metastatic renal cell carcinoma: a phase I dose escalation trial

**DOI:** 10.1186/s13014-017-0893-x

**Published:** 2017-09-22

**Authors:** Katrien De Wolf, Sylvie Rottey, Karim Vermaelen, Karel Decaestecker, Nora Sundahl, Lizzy De Lobel, Els Goetghebeur, Gert De Meerleer, Nicolaas Lumen, Valérie Fonteyne, Daan De Maeseneer, Piet Ost

**Affiliations:** 1 Department of Radiation-Oncology, University Hospital Ghent, De Pintelaan 185, 9000 Ghent, Belgium; 2 Immuno-Oncology Network Ghent (ION Ghent), Ghent, Belgium; 3 Department of Medical Oncology, University Hospital Ghent, De Pintelaan 185, 9000 Ghent, Belgium; 4 Cancer Research Institute Ghent (CRIG Ghent), Ghent, Belgium; 5 Department of Internal Medicine, University Hospital Ghent, De Pintelaan 185, 9000 Ghent, Belgium; 6 Department of Urology, University Hospital Ghent, De Pintelaan 185, 9000 Ghent, Belgium; 7 Department of Applied Mathematics, Computer Science and Statistics Ghent University, Ghent, Belgium; 8 Department of Radiation-Oncology, University Hospital Leuven, Herestraat 49, 3000 Leuven, Belgium

**Keywords:** Tyrosine kinase inhibitors, Stereotactic body radiotherapy, Renal cell carcinoma, Immune monitoring

## Abstract

**Background:**

The primary objective was to determine maximum tolerated radiation dose in patients with metastatic renal cell carcinoma on pazopanib treatment.

**Methods:**

Treatment-naïve patients received pazopanib according to standard of care. Stereotactic body radiotherapy (SBRT) was delivered concurrently to the largest metastatic lesion at day 8, 10 and 12. SBRT doses were escalated in 3 dose levels (24 Gy/3, 30 Gy/3 and 36 Gy/3). Dose level was assigned using Time-to-Event Continual Reassessment Method with the target dose-limiting toxicity rate set to 0.25.

**Results:**

Thirteen patients were included. One patient experienced dose limiting toxicity (DLT) at dose level 3 (grade 4 hypoglycemia). Maximum tolerated dose was not reached with a recommended dose of 36 Gy/3 having a probability of DLT of 11%. One-year local control was 83% (95% confidence interval 61–100) and 1-year progression-free survival was 28% (95% confidence interval 1–55).

**Conclusions:**

SBRT in combination with pazopanib is well tolerated with good local control and response rates outside the radiation field.

**Trial registration:**

This trial was retrospectively registered on clinicaltrials.gov(NCT02334709) on January 6th, 2015.

**Electronic supplementary material:**

The online version of this article (10.1186/s13014-017-0893-x) contains supplementary material, which is available to authorized users.

## Background

Renal cell carcinoma (RCC) presents with metastatic disease in about 30% of patients, while another 30% of patients will ultimately develop metastases [1, 2]. Tyrosine kinase inhibitors (TKIs) targeting vascular endothelial growth factor receptor (VEGFR) are currently considered the mainstay treatment for metastatic patients in first line [3]. Nevertheless, durable responses are rare and most patients eventually develop progressive disease [4, 5]. More recently, PD-1/PD-L1 (programmed cell death ligand) targeting agents, especially nivolumab, have shown durable responses, but only in a minority of patients [6]. Therefore, new therapeutic approaches are needed to improve the number of patients benefiting from durable disease control. The combination of TKIs with high-dose radiation is a promising approach to increase response rate. Preclinical combination studies suggest synergistic radio-sensitizing effects. In addition, both treatments elicit antitumor immune responses making the tumor more susceptible to efficient elimination by immune cells [7]. We hypothesized that the combination of pazopanib (Votrient, Novartis), a first-line TKI, and high-dose radiotherapy could demonstrate superior efficacy compared to either treatment in monotherapy. Although high-dose radiotherapy can be delivered safely by making use of stereotactic body radiotherapy (SBRT) and the safety profiles of TKIs are well studied [8, 9], their concurrent administration may potentially exacerbate adverse events (AEs).

Our primary objective was to determine the maximum tolerated dose (MTD) of SBRT in combination with a fixed dose of pazopanib in patients with metastatic clear cell RCC (ccRCC). Secondary end points included objective response of the non-irradiated lesions, local control, and progression-free survival (PFS). An exploratory endpoint was to assess immunologic responses using peripheral blood samples.

## Methods

### Patients

Patients diagnosed with metastatic ccRCC and having at least 3 extracranial measurable lesions per Response Evaluation Criteria in Solid Tumors (RECIST v1.1) [10] for soft tissue disease or MD Anderson (MDA) criteria [11] for bone lesions were enrolled. Patients were eligible if they had histological confirmed ccRCC, and the presence of measureable disease on whole body imaging by computed tomography (CT). All patients underwent a cytoreductive nephrectomy at least 6 weeks prior to inclusion. Other key eligibility criteria included a Karnofsky Performance Status >60, and adequate organ and bone marrow function, which were defined as absolute neutrophil count greater than 1.5 × 10^6^/L, hemoglobin greater than 9 g/dL, platelet count greater than 100 × 10^9^/L, PT or INR less than 1.2 times the upper limit of normal, aPTT less than 1.2 times the upper limit of normal, total bilirubin less than 1.5 times the upper limit of normal, alanine aminotransferase (ALT) and aspartate aminotransferase (AST) less than 2.5 times the upper limit of normal, and serum creatinine less than 1.5 mg/dL.

Patients were excluded if they had a history of prior radiotherapy interfering with SBRT. Other key exclusion criteria were uncontrolled central nervous system metastases at baseline, severe or active comorbidity likely to impact on the advisability of dose intensified SBRT and uncontrolled intercurrent illness defined as significant gastrointestinal, cardiovascular or respiratory abnormalities. All patients gave informed consent before enrollment.

### Study design and treatment

This is a phase I, non-randomized study (ClinicalTrials.gov identifier: NCT02334709). From February 2014 until April 2016 13 patients were enrolled. Dose escalation of SBRT was performed while administrating a standard fixed dose of pazopanib (800 mg orally once daily). Pazopanib doses could be modified at the treating physician’s discretion, according to tolerance. SBRT was administered to the largest metastatic lesion in 3 fractions on alternate days concurrently with the second week of pazopanib treatment (start day 8). A starting SBRT dose level of 24 Gy in 3 fractions was chosen for the study based on its safety profile [12]. The total dose was delivered in 3 separated fractions (>48 h and <96 h between fractions). The total dose was escalated in 3 dose levels: 24 Gy in 3 fractions of 8 Gy, 30 Gy in 3 fractions of 10 Gy and 36 Gy in 3 fractions of 12 Gy. No higher dose escalation was planned. Dose escalation was designed with use of the time-to-event continuous reassessment method (TITE-CRM). Dose reassessment occurred for each patient that entered the trial. Additional file 1: Table S1 shows a general scheme of the trial.

All patients underwent a CT simulation in supine position with 3 mm CT slice thickness through the metastatic site to be treated. The planning CT covered the target and all organs at risk. Support devices to increase patient comfort were chosen depending on the target localization. Lung and liver tumor sites were simulated with 4D–CT, taking into account breathing. The gross tumor volume (GTV) was defined as gross tumor on CT and/or magnetic resonance imaging (MRI). No clinical target volume (CTV) was delineated. Planning target volume (PTV) was defined as an expansion from GTV to account for organ motion and setup error. Margins depended on the site irradiated with 2 mm margins for bony lesions and 5 mm for other sites. In case of overlap between organ at risk (OAR) and PTV, a PTV_optim was created by subtracting the OAR from the PTV volume. This PTV_optim was used to prescribe the dose instead of the PTV. A Planning Organ at Risk Volume (PRV) expansion of 2 mm was added to OARs and dose constraints applied to this PRV. Dose constraints for OAR were in accordance with the recommendations from the report of the American Association of Physicists in Medicine (AAPM) task group 101 [13]. If a dose constraint could not be achieved due to overlap of the target with an OAR, the target coverage was compromised in order to meet the OAR constraint.

Treatment was prescribed to the periphery of the target (80% of the dose covered 90% of the PTV). Treatment was delivered with static or rotational IMRT with 6–18 MV photons of a linear accelerator using cone-beam CT set-up at each fraction and on-line correction of patient’s position.

### End points and assessments

The primary objective was to assess the safety of the combination of pazopanib and SBRT. Patients were monitored for toxicity bi-weekly during the first 3 months of treatment through physical examination and routine safety laboratory studies. AEs were based on assessments by investigators of patients treated between the start of pazopanib and 90 days after the last radiotherapy fraction. AEs and clinical laboratory tests were graded using the National Cancer Institute Common Terminology Criteria for Adverse Events (CTCAE), version 4.0. The MTD was defined as the dose that was associated with dose-limiting toxicity (DLT) in 25% of patients. DLTs were defined as any of the following treatment-related events that occurred after the first fraction of SBRT: any grade 4–5 metabolic or hematologic toxicity and any grade 3–5 non-hematologic toxicity possibly related to SBRT. Toxicities observed before the start of SBRT were not considered DLTs. Grade 3 metabolic or hematologic toxicities were considered expected events with pazopanib and were not considered SBRT related.

Secondary end points included objective response of the non-irradiated lesions, local control, and PFS. Objective responses were assessed using RECIST v1.1 for soft tissue disease on contrast enhanced CT-scans of thorax and abdomen and were carried out on day 91 and every 3 months thereafter. For the evaluation of bone lesions, the MDA criteria were used. Local failure was defined as an increase in size by ≥20% according to RECIST v1.1 or MDA criteria. PFS was defined as the interval between the start of pazopanib and the earliest date of disease progression or death due to any cause. An exploratory endpoint was to assess immunologic responses using peripheral blood samples.

### Peripheral blood mononuclear cells isolation

Venous blood was drawn using 9 mL EDTA tubes at baseline, before the start of SBRT and at day 91. Peripheral blood mononuclear cells (PBMCs) were isolated by centrifugation on a Ficoll-Hypaque gradient (GE Healthcare, Uppsala, Sweden) within 4 h. The PBMCs were cryopreserved until analysis.

### Flow cytometry

Myeloid derived suppressor cells (MDSCs) were characterized by the CD45+ CD16- CD11b + phenotype, monocytic MDSCs are CD14+ S100A9+ CD124+, granulocytic MDSCs are CD14-CD33 + CD15+. Dendritic cells (DCs) were characterized by the CD45+ lineage- phenotype, plasmacytoid DCs were CD123 + BDCA2+ BDCA3- BDCA1- and myeloid DCs were divided into CD123- BDCA2- BDCA1+ and BDCA3+ cells. Regulatory T cells (Tregs) were defined as CD3+ CD4+ CD25+ FoxP3+ and cytotoxic T-cells as CD3+ CD8+ cells. T helper (Th) subsets were divided into CD3+ CD4+ CD45R0+ memory and CD45RO- naïve Th cells. Th cells were further divided into CXCR3+ Th1 cells, CRTH2+ Th2 cells and CCR6+ Th17 cells. All antibodies used in this study were fluorescently conjugated mouse anti-human monoclonal antibodies. For intracellular staining, PBMCs were fixed and permeabilized using Live/dead® fixable aqua dead cell stain (BD Biosciences) after surface staining, and then stained with mouse anti-human monoclonal antibodies against CTLA-4, PD-1 PE-Texas Red, Lag3 PE-Cy7, Tim3 FITC and FoxP3 APC antibodies. Flow cytometry data were analyzed using FlowJo software (Tree Star Inc., Ashland, OR, USA). Thresholds for signal background were set using isotype and fluorescence-minus-one (FMO) controls, as appropriate. Additional file 1: Figures S2-S5 depict the representative gating strategies.

### Statistical considerations

A TITE-CRM [14] was used to locate the MTD. Dose reassessment occurred for each patient that entered the trial. By making use of weights, staggered entrance of the patients in the trial was allowed. The target probability for the MTD was set at 0.25. Based on simulations, a sample size of 21 was set. Efficacy data were analyzed according to the intention-to-treat principle. The Kaplan-Meier method was used to estimate local control and PFS. For immune monitoring, median values between 2 groups were compared by the Mann-Whitney U-test, between ≥2 groups with Kruskall-Wallis testing. For the evaluation of immunological markers over time, the Friedman test was used. To evaluate correlations, Spearman correlation coefficients were calculated. All statistical analyses were performed using SPSS 24.0 (SPSS Inc., Chicago, IL, USA) and a *P*-value less than 0.05 was considered statistically significant.

## Results

### Patients

Thirteen patients were enrolled between February 2014 and April 2016. Table 1 summarizes patient and disease characteristics at time of SBRT.

**Table 1:** Demographics and baseline characteristics

	*n* (%)
Sex	
Male	7 (54)
Female	6 (46)
Median age (y)	66 (range 48–72)
Karnofsky Performance Status	
100	3 (23)
90	8 (62)
80	2 (15)
Heng criteria	
0	6 (46)
1	4 (31)
2	2 (15)
Unknown	1 (8)
MSKCC criteria	
0	4 (31)
1	7 (54)
2	0 (0)
Unknown	2 (15)
Prior radiotherapy	
No	9 (69)
Yes	4 (31)
Number of organs involved	
1	4 (31)
2	7 (54)
3	2 (15)
SBRT treatment site	
Lung	5 (38)
Bone	2 (15)
Lymph node	2 (15)
Pancreas	1 (8)
Soft tissue mass	2 (15)
Liver	1 (8)

*SBRT* stereotactic body radiotherapy, *MSKCC* Memorial Sloan-Kettering Cancer Center

### Adverse events

After 13 patients were enrolled, an interim evaluation was done, prompted by newly available TKIs, nivolumab and competing trials with immunotherapeutic agents likely to hamper further enrolment into this study. No DLTs were noted at dose levels 1 or 2 (24 Gy and 30 Gy). Of 8 patients at dose level 3 (36 Gy), 1 patient with a history of diabetes mellitus type 2, who was irradiated at a mediastinal lesion, experienced a DLT consisting of grade 4 hypoglycemia. No increased toxicity inside the radiation fields was seen. The grade 4 hypoglycemia resolved completely after adjusting insulin treatment.

The interim evaluation estimated the probability of having a DLT at 11%. Given this result, continuing to 21 patients, would yield an estimated DLT rate below 25% and hence an unchanged conclusion as long as the final number of DLTs stayed strictly below 5. The currently estimated chance for this is >99%. The study was therefore closed after 13 patients. The MTD was therefore not reached and 36 Gy/3, having a probability of DLT of 11%, was selected as the recommended dose for future phase II trials (Table 2). The vast majority of AEs were grade 1 and grade 2 (Table 3). Grade 3 to 4 pazopanib-related AEs occurred in 38% of patients. The most common grade 3 or 4 AEs were hypoglycemia, increased ALT and increased AST in 1 of 13 patients and hypertension in 3 of 13 patients. Six patients (46%) needed a dose reduction of pazopanib due to AEs, 1 of 4 patients in dose level 1 (reduced to 400 mg daily), 1 patient in dose level 2 (reduced to 600 mg daily) and 4 of 8 patients in dose level 3 (reduced to 400 mg daily in 3 patients and to 600 mg daily in 1 patient). There were no patients who discontinued neither pazopanib treatment nor SBRT due to AEs. No fatal AEs were reported.

**Table 2:** Maximum tolerated dose

Level	Dose	Number treated	Number of DLTs	Probability of DLT
1	3 × 8 Gy	4	0	0.05
2	3 × 10 Gy	1	0	0.08
3	3 × 12 Gy	8	1	0.11

**Table 3:** Treatment-related adverse events

	24 Gy	30 Gy	36 Gy	ALL
	*n* = 4	*n* = 1	*n* = 8	*n* = 13
Laboratory abnormalities, any grade				
Anemia	1	0	2	3
Leucopenia	1	0	2	3
thrombocytopenia	2	0	7	9
Lymphocytopenia	3	1	4	8
Hypoglycemia	2	1	4	7
Increased alanine aminotransferase	4	1	4	9
Increased aspartate aminotransferase	3	1	4	8
Increased alkaline phosphatase	2	1	1	4
Increased creatinine	0	1	3	4
Hypothyroidism	1	0	4	5
Hyperkalemia	3	1	1	5
Adverse events, any grade				
Fatigue	4	1	6	11
Insomnia	3	0	1	4
Anorexia	0	0	3	3
Weight loss	0	0	4	4
Dysgeusia	1	0	8	9
Dry mouth	1	1	2	4
Nausea	1	0	4	5
Vomiting	1	0	3	4
Dyspnea	1	0	6	7
Hypertension	3	1	5	9
Peripheral edema	2	0	1	3
Dry skin	1	0	2	3
Changes in hair color	0	1	2	3
Hand foot syndrome	1	0	2	3
Laboratory abnormalities, grade 3–4				
Hypoglycemia	0	0	1	1
Increased alanine aminotransferase	0	0	1	1
Increased aspartate aminotransferase	0	0	1	1
Adverse events, grade 3–4				
Hypertension	1	0	2	3

### Efficacy

**Fig. 1:**
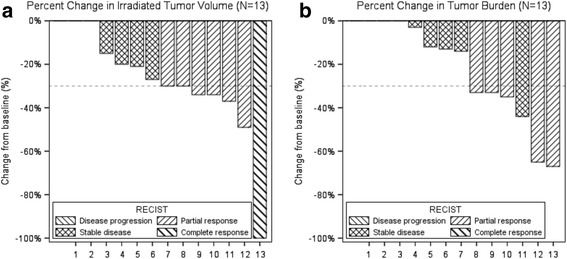
Local control of irradiated lesions and distant control of non-irradiated lesions. **a**: Greatest percentage change in irradiated tumor volume. Complete response, partial response, stable disease and disease progression were assessed as per RECIST 1.1 or as per MDA criteria for bone lesions. Two patients did not have any change in irradiated tumor volume. **b**: Greatest percentage change in tumor volume of non-irradiated target lesions. Complete response, partial response, stable disease or disease progression were assessed as per RECIST 1.1 or as per MDA criteria for bone lesions. Three patients did not have any change in non-irradiated target lesions, one patient had a decrease in non-irradiated tumor burden yet had progressive disease due to a new lesion (this is not added to the tumor burden calculation as per RECIST 1.1)

We noted a complete local response in 1 of 13 irradiated lesions (8%), partial response (PR) in 6 of 13 irradiated lesions (46%), and stable disease (SD) in 6 of 13 irradiated lesions (46%) as best response (Fig. 1a and Additional file 1: Figure S1a). Median duration of local control was not reached and 1-year local control was 83% (95% confidence interval (CI) 61–100). Assessment of responses outside the radiation field revealed that 5 of 13 patients (38%) developed a PR, 7 patients (54%) had SD and 1 patient (8%) had progressive disease (PD) as best response (Fig. 1b and Additional file 1: Figure S1b), making the objective response rate (ORR) 38%. Median PFS was 6.7 months (95% CI 3–10) and 1-year PFS was 28% (95% CI 1–55). Median follow-up was 10.9 months. No patients were lost to follow up.

### Systemic immune changes during treatment

**Fig. 2:**
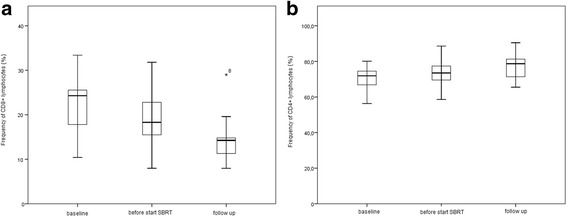
Frequency of CD8+ and CD4+ lymphocytes during treatment. **a**: Boxplot comparing the frequency of CD8+ lymphocytes at baseline, before start of SBRT and at the first evaluation visit. **b**: Boxplot comparing the frequency of CD4+ lymphocytes at baseline, before start of SBRT and at the first evaluation visit

PBMCs from 11 of 13 patients were collected for immune monitoring. We observed a decrease in the frequencies of CD8+ lymphocytes (*P* = 0.027) and an increase in CD4+ lymphocytes (*P* = 0.014) during treatment (Fig. 2).

### Link between T cell subsets and prognosis

**Fig. 3:**
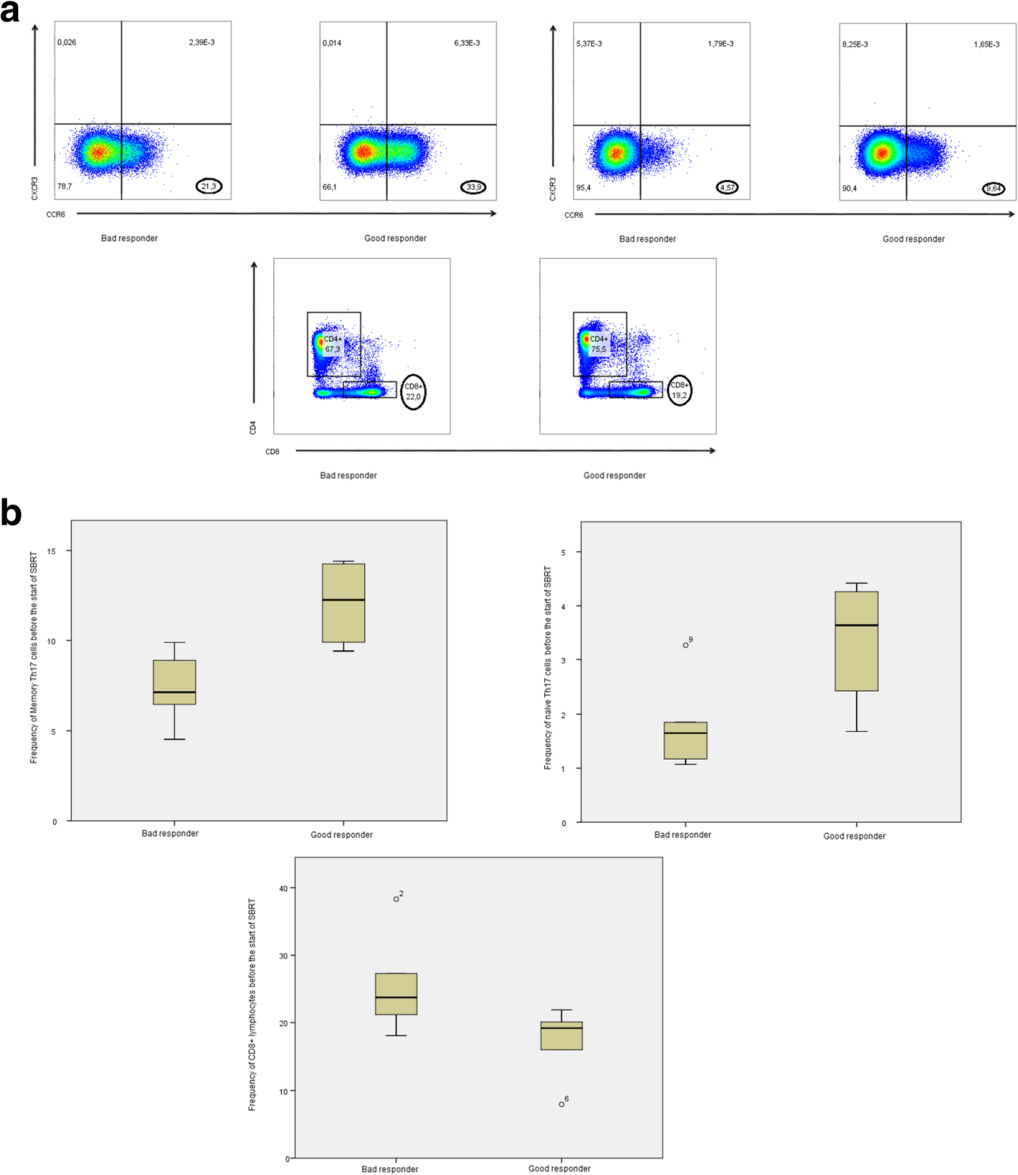
Frequency of cells before the start of SBRT between good and bad responders. **a** Frequency of Memory Th17 cells, Naive Th17 cells and CD8+ lymphocytes before the start of SBRT in 1 bad responding study patient compared to 1 good responding study patient. **b** Boxplot comparing the frequency of Memory Th17 cells, Naive Th17 cells and CD8+ lymphocytes before start of SBRT between bad responding and good responding patients

Patients were divided into good responders and bad responders based on the median PFS of 8.4 months of pazopanib in monotherapy [8]. We compared PBMCs from good responders and bad responders. We observed lower frequencies of CD8+ lymphocytes after the first week of pazopanib treatment in good responders as compared to bad responders (*P* = 0.036). We also observed a shift in T cell subsets. The frequency of memory Th17 cells after the first week of pazopanib treatment was significantly higher in good responders compared to bad responders (*P* = 0.019). A similar though non-significant difference was also observed for naive Th17 cells (*P* = 0.054) (Fig. 3). We also observed a non-significant increase in CTLA-4 expression on Tregs after one week of pazopanib treatment in patients with a good prognosis (*P* = 0.063).

## Discussion

Until recently, radiotherapy in metastatic RCC was primarily used to palliate symptomatic metastases [3] as RCC has been traditionally considered a radiation-resistant tumor. Although RCC might be resistant to conventional fractionated radiation, recent evidence suggested the opposite for high-dose radiotherapy [15]. By making use of SBRT, it is possible to safely deliver high radiation doses and SBRT for metastatic RCC has been associated with impressive 1-year local control rates ranging from 71% - 100% [15–22]. This may be due to either the destruction of tumor microvasculature and/or the induction of antitumor immune responses associated with SBRT (6, 13). TKIs used as first-line therapy in metastatic RCC also have the potential to interact with the immune system. For example, sunitinib, the most-studied TKI in the treatment of RCC, has important immunostimulatory capacities [23, 24]. The immunomodulatory capacities of pazopanib are less well studied, but the effects may be similar. Combined treatment of SBRT and TKIs might therefore increase the antitumor activity of both treatments [7]. We hypothesized that the combination of pazopanib and SBRT could increase response rates. The safety of the combination of pazopanib with conventional radiotherapy has already been investigated [25, 26]. However, there are only limited retrospective cases reported on SBRT and pazopanib [27]. Since their concurrent administration could potentially exacerbate AEs, a prospective phase I dose-escalation trial was conducted. In our trial the MTD was not reached. In dose level 3 (36 Gy), 1 DLT of grade 4 hypoglycemia was reported in a patient with a history of diabetes mellitus type 2, who was irradiated on a mediastinal lesion. Importantly, no increase in radiation-induced toxicity was observed. The grade 4 hypoglycemia resolved completely after adjusting insulin treatment. Because it was assumed that SBRT could also potentially exacerbate pazopanib-related AEs [26, 27], all grade 4–5 metabolic or hematologic toxicities were defined as DLT, regardless of the radiotherapy field. In retrospect, this definition was possibly too strict. In the dose level 3 group, a slightly higher rate of thrombocytopenia was observed, though not dose limiting. We investigated whether a higher incidence of bony lesions or a higher radiation dose on the bone marrow in this group could be the cause, yet only 2 patients in dose level 3 and 1 patient in dose level 1 had bony lesions. In both groups only 1 patient was irradiated on a bony lesion. Therefore, the seemingly increase in thrombocytopenia in dose level 3 was probably due to chance since the number of patients in dose levels 1 and 2 were small.

This trial also provides evidence on antitumor activity of the combination treatment. All patients in our trial initially achieved local control and 1-year local control rates were comparable to those of SBRT in monotherapy [16–22]. However, these data are mostly derived from patients with limited metastatic disease or inoperable localized disease, instead of patients with more extensive metastatic disease enrolled in our study. Regarding responses outside the radiation field, the ORR was 38% with 5 of 13 patients developing a PR. These data are comparable to the ORR of pazopanib in monotherapy [8]. To study the underlying immunomodulatory effects of the combination treatment, we analyzed PBMCs derived at fixed intervals during treatment. Since the number of patients in the trial was limited, the presented data should be interpreted as exploratory. Frequencies of memory Th17 cells were significantly higher in good responders as compared to bad responders. The role of Th17 cells in cancer is controversial with both tumor promoting and tumor suppressing functions being reported [28–30]. This may rely on the existence of regulatory vs. pathogenic Th17 subpopulations, the latter subset being involved in auto-immune tissue damage and tumor rejection [31, 32]. Further contributing to the tumor suppressing function of Th17 cells is the production of the chemokines CXCL9 and CXCL10, which facilitates the recruitment of CD8+ T cells into the tumor [33]. The present study also revealed that lower frequencies of CD8+ T cells were associated with good prognosis, which may be due to the majority of CD8+ T cells being recruited into the tumors.

## Conclusion

In our phase I study, the MTD was not reached, resulting in a recommended dose of 36Gy/3 for combination with pazopanib. To the author’s knowledge, this is the first report demonstrating that SBRT in combination with pazopanib is well tolerated. Local control and response rates outside the radiation field were good but seemed not to be superior when compared to SBRT or pazopanib in monotherapy. The combination of SBRT with pazopanib and investigation of CD8+ T cells and Th17 cells as a predictor of response warrant further study.

## Additional file

Additional file 1: Table S1. General scheme of the trial. **Figure S1.** Change in volume of irradiated lesions and non-irradiated lesions as a function of time. **Figure S2.** Gating strategy: Monocytes and lymphocyte subsets. **Figure S3.** Gating strategy: Dendritic cell subsets. **Figure S4.** Gating strategy: T cell subsets. **Figure S5.** Gating strategy: Immune suppressive markers on T cells. (DOCX 610 kb)

## Abbreviations

AAPM: American Association of Physicists in Medicine
AE: Adverse event
ALT: Alanine aminotransferase
AST: Aspartate aminotransferase
ccRCC: Clear cell renal cell carcinoma
CI: Confidence interval
CT: Computed tomography
CTCAE: Common Terminology Criteria for Adverse Events
CTV: Clinical target volume
DC: Dendritic cell
DLT: Dose limiting toxicity
FMO: Fluorescence-minus-one
GTV: Gross tumor volume
MDA: MD Anderson criteria
MDSC: Myeloid derived suppressor cells
MRI: Magnetic resonance imaging
MTD: Mean tolerated dose
OAR: Organ at risk
ORR: Objective response rate
PBMC: Peripheral blood mononuclear cell
PD: Progressive disease
PD-1: Programmed cell death 1
PD-L1: Programmed cell death ligand 1
PFS: Progression-free survival
PR: Partial response
PTV: Planning target volume
RCC: Renal cell carcinoma
RECIST: Response Evaluation Criteria in Solid Tumors
SBRT: Stereotactic body radiotherapy
SD: Stable disease
Th: T helper cell
TITE-CRM: Time-to-Event Continual Reassessment Method
TKI: Tyrosine kinase inhibitors
Treg: Regulatory T cell
VEGFR: Vascular endothelial growth factor receptor
