# Supplementary material for: Combined high dose radiation and pazopanib in metastatic renal cell carcinoma: a phase I dose escalation trial
Source: Radiat Oncol. 2017 Sep 22;12:157. doi: 10.1186/s13014-017-0893-x (PMC5610443; doi:10.1186/s13014-017-0893-x)

**Supplementary Information**

Table S1. General scheme of the trial

| **Treatment modality** | **Run - in period** | **Study period** | | | **Evaluation period** |
| --- | --- | --- | --- | --- | --- |
|  |  |  | | |  |
|  | **d1** | **d8** | **d10** | **d12** | **d84-98** |
| pazopanib | start | continuous administration | | | |
|  |  | SBRT | SBRT | SBRT |  |
| SBRT |  |  |  |  |  |

| **Dose level** | **Dose of SBRT (total daily dose)** | **Dose of SBRT  (total dose)** | **Minimum number of patients** |
| --- | --- | --- | --- |
| 1 | 8 Gy | 24Gy | According to TITE-CRM |
| 2 | 10 Gy | 30 Gy | According to TITE-CRM |
| 3 | 12 Gy | 36 Gy | According to TITE-CRM |

SBRT=stereotactic body radiotherapy, TITE-CRM= time-to-event continuous reassessment method

Figure S1: Change in volume of irradiated lesions and non-irradiated lesions as a function of time


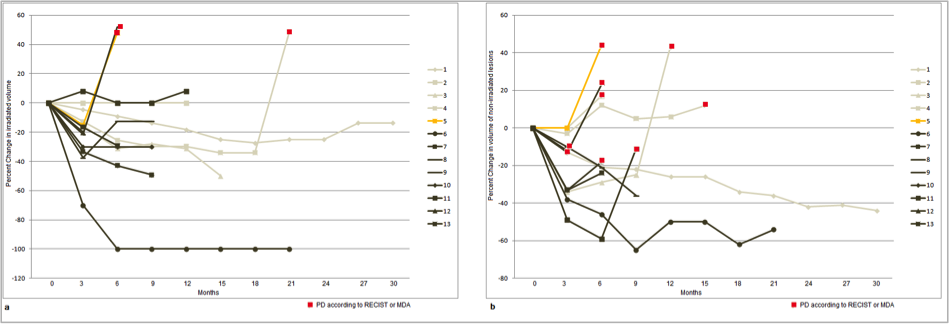
Figure S1A: Greatest percentage change in irradiated tumor volume as a function of time. Patients in dose level 1 are represented by the grey lines, patients in dose level 2 by the yellow line and patients in dose level 3 by the black lines. Progressive disease was defines per RECIST 1.1 or per MDA criteria for bone lesions. Figure S1B: Greatest percentage change in tumor volume of non-irradiated target lesions as a function of time.

Figure S2: Gating strategy: Monocytes and lymphocyte subsets


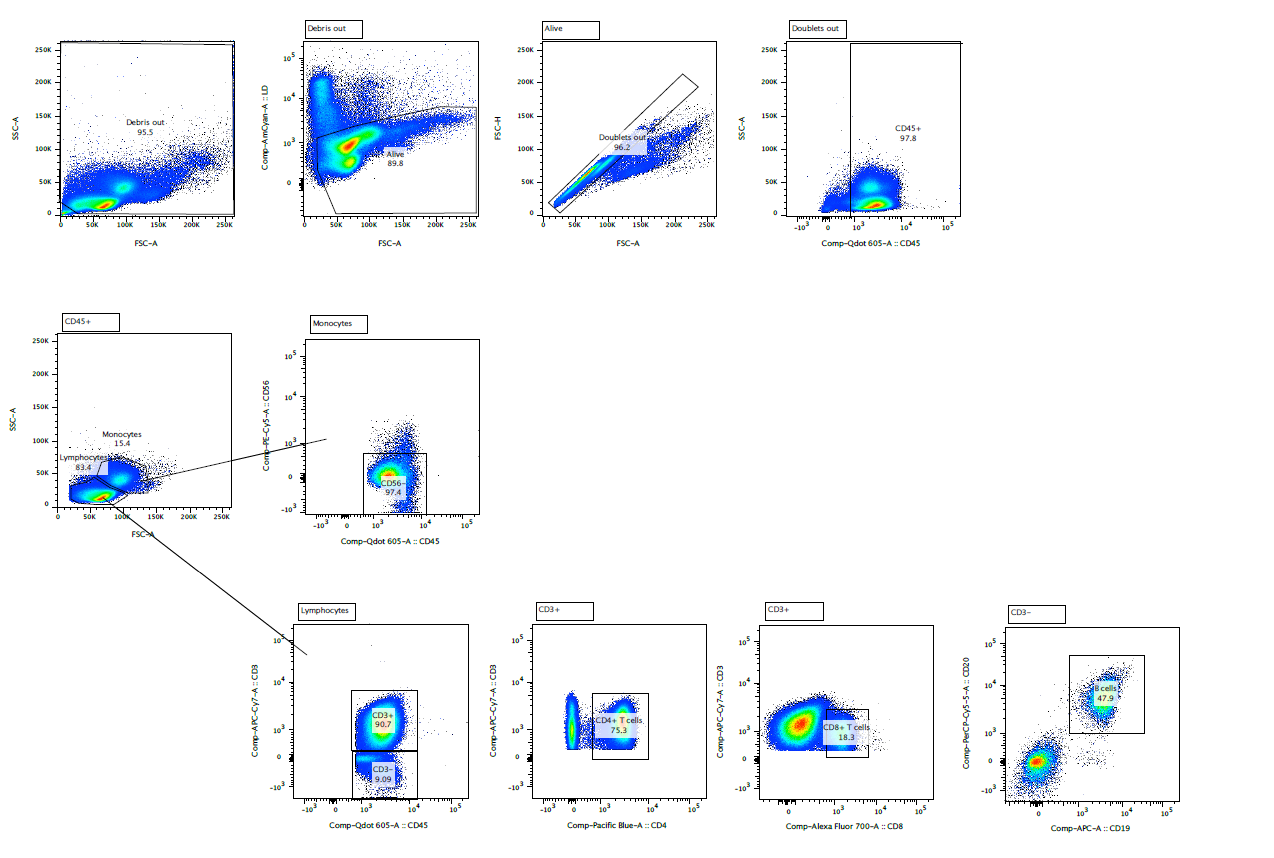


Figure S3: Gating strategy: Dendritic cell subsets


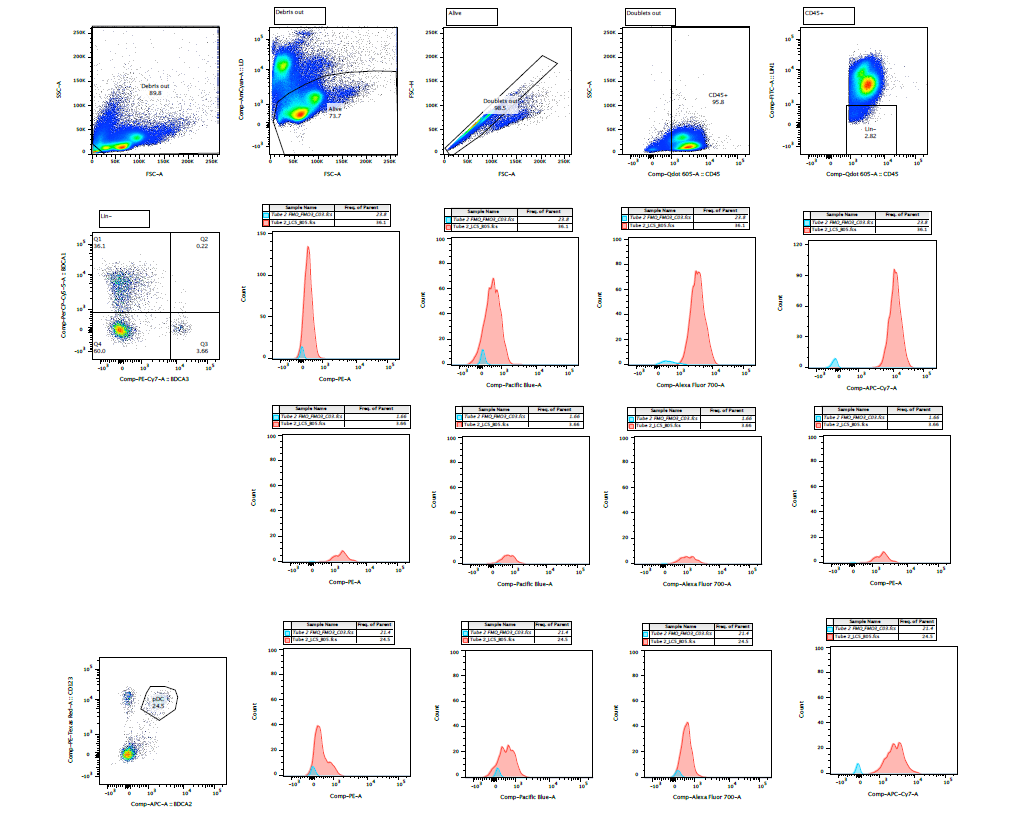


Figure S4: Gating strategy: T cell subsets


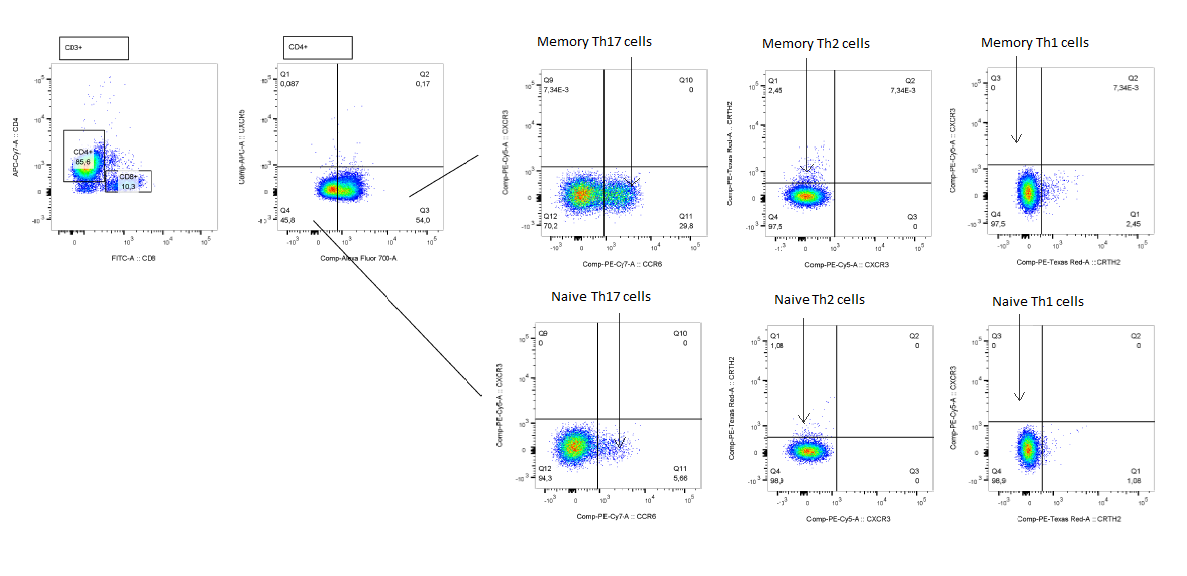


Figure S5: Gating strategy: Immune suppressive markers on T cells


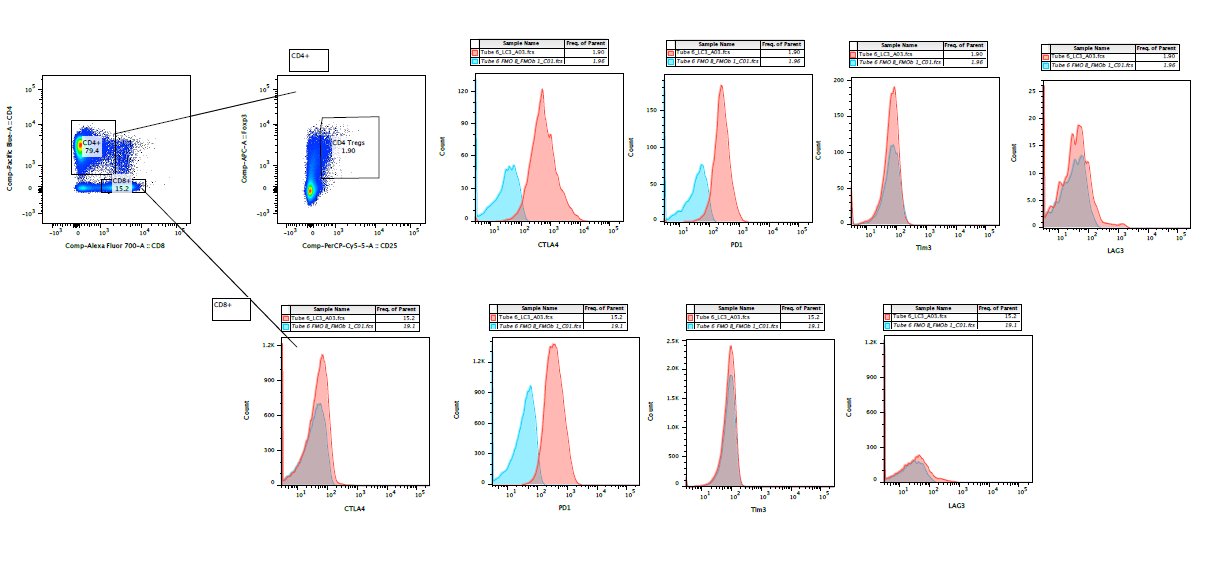

Supplement: Additional file 1: Table S1. — General scheme of the trial. Figure S1. Change in volume of irradiated lesions and non-irradiated lesions as a function of time. Figure S2. Gating strategy: Monocytes and lymphocyte subsets. Figure S3. Gating strategy: Dendritic cell subsets. Figure S4. Gating strategy: T cell subsets. Figure S5. Gating strategy: Immune suppressive markers on T cells. (DOCX 610 kb) [file 13014_2017_893_MOESM1_ESM.docx]
